# Transplantation of Human Umbilical Cord Mesenchymal Stem Cells-Derived Neural Stem Cells Pretreated with Neuregulin1β Ameliorate Cerebral Ischemic Reperfusion Injury in Rats

**DOI:** 10.3390/biom12030428

**Published:** 2022-03-10

**Authors:** Qiu-Yue Zhai, Yu-Qian Ren, Qin-Shuai Ni, Zhen-Hua Song, Ke-Li Ge, Yun-Liang Guo

**Affiliations:** 1 Institute of Cerebrovascular Diseases, Medical Research Center, The Affiliated Hospital of Qingdao University, Taishan Scholars Construction Project Excellent Innovative Team of Shandong Province, Qingdao 266003, China; 2018010040@qdu.edu.cn (Q.-Y.Z.); 2019020984@qdu.edu.cn (Y.-Q.R.); 2020021111@qdu.edu.cn (Q.-S.N.); 2 Institute of Pharmacology, Qingdao Medical College, Qingdao University, Qingdao 266021, China; songzh@qdu.edu.cn; 3 Institute of Integrative Medicine, Qingdao Medical College, Qingdao University, Qingdao 266021, China; gekeli@qdu.edu.cn

**Keywords:** neural stem cells, neuregulin1β, middle cerebral artery occlusion/reperfusion, ferroptosis, transplantation

## Abstract

Ischemic stroke is a common cerebrovascular disease and recovering blood flow as early as possible is essential to reduce ischemic damage and maintain neuronal viability, but the reperfusion process usually causes additional damage to the brain tissue in the ischemic area, namely ischemia reperfusion injury. The accumulated studies have revealed that transplantation of exogenous neural stem cells (NSCs) is an ideal choice for the treatment of ischemia reperfusion injury. At present, the source and efficacy of exogenous NSCs after transplantation is still one of the key issues that need to be resolved. In this study, human umbilical cord mesenchymal stem cells (hUC-MSCs) were obtained and induced into NSCs byadding growth factor and neuregulin1β (NRG1β) was introduced during the differentiation process of NSCs. Then, the rat middle cerebral artery occlusion/reperfusion (MCAO/R) models were established, and the therapeutic effects were evaluated among groups treated by NRG1β, NSCs and NSCs pretreated with 10 nM NRG1β (NSCs-10 nM NRG1β) achieved through intra-arterial injection. Our data show that the NSCs-10 nM NRG1β group significantly improves neurobehavioral function and infarct volume after MCAO/R, as well as cerebral cortical neuron injury, ferroptosis-related indexes and mitochondrial injury. Additionally, NSCs-10 nM NRG1β intervention may function through regulating the p53/GPX4/SLC7A11 pathway, and reducing the level of ferroptosis in cells, further enhance the neuroprotective effect on injured cells.

## 1. Introduction

Ischemic stroke is the second most common cause of death and disability in the world, and with the aging of the world’s population, the incidence of which is increasing year by year [1]. It has been reported that ischemic stroke is a phenomenon in which the lack of blood supply in the brain leads to the insufficient supply of oxygen and glucose for the survival of neurons, thus changing the microenvironmental homeostasis of neurons and ultimately leading to cell death [2]. At present, the intravenous thrombolysis and intravascular thrombectomy are commonly used to treat patients with ischemic stroke [3,4]. It is generally believed that blood supply restoration is essential to save ischemic tissue and reduce the rate of disability and mortality in ischemic stroke patients. However, reperfusion also causes a series of cellular stresses, including accumulation of reactive oxygen species (ROS), autophagy activation, inflammatory response, the release of apoptotic factors and mitochondrial dysfunction, which often aggravate neurovascular injury, resulting in the destruction of the blood–brain barrier and the death of nerve cells, leading to additional damage to brain tissue, namely ischemia reperfusion injury [5,6,7].

Due to the harmful effects of the ischemia microenvironment and the limited regeneration ability of endogenous neural cells, many ischemic stroke patients lack an appropriate immune microenvironment as well as enough nerve cells to complement and repair damaged brain tissue, and eventually produce a permanent partial loss of nerve tissue and brain nerve function defect after regional blood flow restoration [8]. Along with the development of stem cells research, disease therapy using stem cells, especially neural stem cells (NSCs), has attracted more and more attention, which provides novel insights for the treatment of neurological diseases including ischemic stroke in the future in clinical. Studies have found that transplantation of exogenous NSCs can significantly repair or replenish damaged tissues, which not only stimulate the production of endogenous NSCs, but also improve the immune microenvironment in the surrounding tissue injury area [9]. However, transplantation therapy with exogenous NSCs still has a long way to go before clinical application, and the ethical issues, as well as therapeutic efficacy and safety, are required to be well addressed. Meanwhile, the source, survival, and differentiation rate of NSCs after transplantation are also key issues to be solved [10]. Therefore, many studies have attempted to use different strategies to increase the therapeutic effect of transplanted cells, such as pretreatment with inflammatory immune factors, and combined strategies with multiple cytokines [11].

It is reported that neuregulin1 (NRG1), a family of epidermal growth factor (EGF), can regulate the growth and development of glial cells, neurons, and cardiomyocytes, also promote the differentiation of neurons and proliferation of glial cells [12,13,14]. Owing to the selective splicing at different parts of transcription products, NRG1 can be divided into α or β type, which has different affinity for different receptors [15]. Studies have demonstrated that NRG1β and its main receptor ErbB4 is widely present in the hippocampus, cerebral cortex, piriform cortex, thalamic reticular nucleus, and ventral midbrain [16]. At the same time, there are growing evidences suggest that NRG1β and ErbB genes are involved in complex brain diseases, and NRG1β has also been reported to prevent brain injury after stroke and play a neuroprotective role in mouse models of Parkinson’s disease, Alzheimer’s disease, and traumatic brain injury [17,18,19,20]. However, whether NRG1β combined with NSCs can improve the therapeutic effect of NSCs has not been reported.

Recent studies have reported that ferroptosis is related to ischemia reperfusion injury [21,22]. First, inhibition of tau protein (the major microtubule-associated protein) can lead to iron accumulation after ischemia, which can lead to the imbalance of iron metabolism and promote the production of ROS, resulting in nucleic acid, proteome, and membrane damage, and ultimately lead to cell death [23]. In addition, Guan et al. suggested that carvone could inhibit ferroptosis by enhancing GPX4 expression, thereby saving hippocampal neuron injury induced by ischemia reperfusion injury [24]. Similarly, Lanet et al. found that the Naotaifang (a kind of Chinese herbal medicine) compound can also reduce neuronal ferroptosis after ischemic stroke through regulating transferrin receptor 1 (TFR1)/divalent metal transporter 1 (DMT1) and SCL7A11/GPX4 pathways [25]. These studies suggest that ferroptosis may be a potential therapeutic target for clinical stroke intervention.

To explore whether the combined action of NRG1β and exogenous NSCs can improve the intervention effect on ischemic stroke reperfusion injury, and whether ferroptosis plays a role in the process, human umbilical cord mesenchymal stem cells (hUC-MSCs) were induced into NSCs by adding growth factors in this study, and NRG1β was added during the induction of NSCs. Then, by using a rat middle cerebral artery occlusion/reperfusion (MCAO/R) model, the intervention effect of NSCs pretreated with NRG1β on MCAO/R injury was evaluated in vivo, and the molecular regulatory mechanism of NRG1β pretreated NSCs during treatment was further explored.

## 2. Materials and Methods

### 2.1. Middle Cerebral Artery Occlusion/Reperfusion (MCAO/R) Model

Adult Male Sprague-Dawley rats (body weight ranging from 230 g to 250 g) used in the study were purchased from the Vital River Laboratory Animal Technology Co., Ltd. (Beijing, China), and the rats were bred in the Experimental Animal Center of Qingdao University. The rats were housed for 7 days before surgery and were accustomed to human handling prior to behavioral testing. The MCAO/R model was established as described previously with minor modifications based on our laboratory [26]. Briefly, the rats were anesthetized by using 2% 2,2,2-Tribromoethanol at a dose of 350 mg/kg through an intraperitoneal injection and were placed on a constant temperature heating pad to maintain a body temperature of 37.0 °C during the MCAO/R surgery. Then, the left common carotid artery (CCA), external carotid artery (ECA) and internal carotid artery (ICA) were exposed via a ventral midline incision. The ECA was ligated with No.4 nylon monofilament, and ICA and CCA were clipped using microvascular arterial clips. A 2.8 silicone-coated nylon was inserted into the ICA from CCA through the ECA stump, and was gently advanced 18 to 20 mm to occlude the middle cerebral artery. After 90 min of MCAO occlusion, the silicone-coated nylon was carefully withdrawn to restore the blood supply. The Sham-operated group underwent a similar surgery but without occlusion and reperfusion. A total of 150 rats were used in this study, 20 rats were selected as the Sham group by random number table method. Next, the remaining 130 rats were used for the MCAO/R model, and the successfully modeled rats were further randomly divided into MCAO/R, MCAO/R + NRG1β, MCAO/R + NSCs, MCAO/R + NSCs-10 nM NRG1β groups, and at least 15 rats in each group were guaranteed to survive. A total of 92 rats survived after surgery in five groups, the survival rate in the Sham, MCAO/R, MCAO/R + NRG1β, MCAO/R + NSCs, MCAO/R + NSCs-10 nM NRG1β groups were 100.00%, 50.00%, 51.61%, 58.82% and 60.61%, respectively. The rats showing no awakening from anesthesia after 90 min of ischemia, died of anaesthesia accident, neurological scores that did not meet the requirements after MCAO, and survival time did not reach 7 days after operation were excluded from the experimental group in this study. One person randomly selected groups and rats in the group at regular times every day, and scored them according to the scoring scale. The process of grasping, putting back and scoring should be handled gently to avoid stimulation and injury to the rat. All experimental procedures on animals were approved by the he Affiliated Hospital of Qingdao University and the Experimental Animal Ethics Committee of Qingdao University (QYFY WZLL 26598, 20210720SD15020211230062).

### 2.2. Preparation of NSCs and Immunofluorescence

hUC-MSCs were obtained from umbilical cord by tissue block culture method, and then they were induced into NSCs after passage cultivation. Specifically, NSCs were induced from hUC-MSCs in vitro for 7 days under the induction medium consisting of DMEM/F12 (Gibco, C11330500BT, Grand Island, NY, USA), 20 ng/mL EGF (PreproTech, AF-100-15-100, Rocky Hill, NJ, USA), 20 ng/mL basic fibroblast growth factor (bFGF, PreproTech, AF-100-18B-50, Rocky Hill, NJ, USA) and 2% B27 supplement (Gibco, 12587010, Grand Island, NY, USA). In the NRG1β pretreatment group, a dose of 10 nM NRG1β (R & D, 396-HB, Minneapolis, MN, USA) was added into the NSCs induction medium during the 7 days from hUC-MSCs to NSCs based on previous studies [27,28,29].

The neurospheres were collected and washed with PBS, then fixed with 4% paraformaldehyde.Next,, neurospheres were permeabilized and then blocked at room temperature for 1 h. Primary antibody SOX2 (1:100, Proteintech, 11064-1-AP, Wuhan, China) and Nestin (1:100, STEMCELL, 60091.1, Seattle, WA, USA) were incubated with at 4 °C overnight. The next day, the fluorescent secondary antibody (1:100, Beyotime, A0423/A0473, Shanghai, China) was incubated, then the nucleu was stained with Hoechst 33,342 (Beyotime, C1025, Shanghai, China).

### 2.3. Cells Transplantation

On the day of transplantation, the neurosphere was prepared in a single-cell suspension with accutase (STEMCELL, 07920, Seattle, WA, USA), which was suspended in sterile phosphate-buffered saline (PBS, Hyclone, SH30256.01, Logan, UT, USA). Intra-arterial (IA) cell grafting was performed as previously described with modifications [30,31]. Briefly, the CCA and ICA was ligated after MCAO/R, then the injection catheter was inserted to the CCA through ECA stump. By releasing the artery clip at the ICA slowly, NSCs and NSCs-10 nM NRG1β (1 × 10^6^) in 100 μL PBS were directly injected into the CCA over the course of 5 min respectively [32], cells transplantation was completed within 10 min. Additionally, Sham and MCAO/R group received the same volume of PBS through same method, and NRG1β at a dose of 2 μg/kg body weight (bw) was given to rats in MCAO/R + NRG1β group. Samples were collected 7 days after transplantation for subsequent studies.

### 2.4. Behavioral Tests

The behavioral tests were performed after MCAO/R (day 0–7) in all experimental animals. Neurological function scores were evaluated by modified neurological severity score (mNSS) as previously described with a few modifications [33,34]. The items of mNSS include muscle tension, motor, sensory, equilibrant and reflex with the total score of 18 (Table 1) [35,36], and the higher scores usually mean more severe neurologic impairment.

### 2.5. Triphenyltetrazolium Chloride (TTC) Staining and Analysis of Infarct Size

Infarct volume was evaluated with TTC staining on the 7th day after transplantation surgery. The rat’s brain was removed carefully and placed in the rat brain matrix for 20 min at −20 °C. The brains were dissected into coronal sections and sliced into 5 sections of 2 mm in thickness, and then they were incubated in 1% TTC (Sigma, T8877, St. Louis, MO, USA) at 37 °C for 20 min. The infarct area remained white, while the normal area of brain was dark red. The volume of the infarct area was measured by Image Pro Plus, and the infarct volume was calculated by the ratio of the volume of the white areas to the whole brain volume.

### 2.6. Nissl Staining

Paraffin sections of brain tissues were performed with Nissl staining solution (Solarbio, G1432, Beijing, China) according to the manufacturer’s instructions. The slices were dewaxed with xylene and immersed in gradient ethanol, then they were treated with Nissl staining solution and immersed in differentiation solution for differentiation. Neurons with round and palely stained nuclei were considered as surviving cells, whereas shrunken cells with pyknotic nuclei were considered as damaged cells. Then, the degree of damaged cells was evaluated by denatured cell index (DCI = degenerated cells/total number of cells × 100%). A Nikon microscope (Nikon, ECLIPSE Ti2, Tokyo, Japan) was used to collect images, and five areas were randomly selected from each group for statistical analysis; a total of 3 replicates were performed.

### 2.7. Measurement of Ferroptosis Index in Brain Tissue

The ischemic cortex limited to the injured region for measurement was obtained from the ischemic side in each group on the 7th day. The obtained tissues were mechanically homogenized with a glass grinder, then the supernatants were collected after centrifuging for further analysis. The levels of Fe^2+^ (Solarbio, BC4355, Beijing, China), ROS (BestBio, BB-470538, Shanghai, China), malondialdehyde (MDA, Beyotime, S0131S, Shanghai, China) and glutathione (GSH, Beyotime, S0053, Shanghai, China) were determined using commercial testing kits according to the manufacturers’ instructions, and the expression value was detected in absorption/emission of specific wavelength with a microplate reader (BioTek, SynergyH1, Winooski, VT, USA).

### 2.8. Transmission Electron Microscope (TEM)

About 1 mm^3^ of ischemic cortex tissue was collected and then steeped in 2.5% glutaraldehyde at 4 °C for 2 days. Then the tissue block was fixed with 1% osmic acid, and gradient dehydrated with acetone, then the samples were embedded in propylene oxide and resin. The samples were then sliced with a Leica ultramicrotome (Leica EMUC7, Wetzlar, Germany) and stained with uranyl acetate and lead citrate, which were observed and taken photographs under HT7700 transmission electron microscope (HITACHI, Tokyo, Japan) finally. Five fields were randomly selected in each group and three times were repeated in each group to calculate the proportion of damaged mitochondria, the proportion of mitochondria in each group = the number of damaged mitochondria/the total number of mitochondria.

### 2.9. Western Blot Analysis

Ischemic cortex limited to the injured region was homogenized in RIPA buffer (Beyotime, P0013C, Shanghai China) on ice and centrifuged at 12,000 rpm for 2 min. The supernatant was collected and protein concentration was measured with BCA kits (CWBIO, CW0014, Beijing, China). Proteins were separated by 10% sodium dodecyl sulphate-polyacrylamide gel electrophoresis, and then were transferred onto polyvinylidene fluoride membranes (PVDF, Millipore, ISEQ00010, USA) Then the membranes were blocked with 5% non-fat milk and incubated with primary antibodies (Table 2) overnight at 4 °C. The secondary antibody of goat anti-rabbit (1:1000, Beyotime, A0208, Shanghai, China) was incubated at room temperature on the next day, and then the targeted membranes were detected with enhanced chemiluminescence (ECL) reagents (Affinity, KF001, Cincinnati, OH, USA). ACTIN was used as a loading control, and the gray value analysis of each protein was performed with Image J software.

### 2.10. Statistical Analysis

Data were presented as the mean ± SD, and the significance analysis was conducted with SPSS 22.0. The differences between groups were determined by one-way ANOVA followed by Dunnett’s, and two-way ANOVA was used to analyses for behaviors testing. The independent experiments were performed at least three times, differences were considered statistically significant when the *p*-value was <0.05 (*) and *p*-value < 0.01 was viewed as highly significant (**).

## 3. Results

### 3.1. Transplantation of hUC-MSCs Derived NSCs Pretreated with NRG1β Ameliorate Neurological Deficits

**Figure 1:**
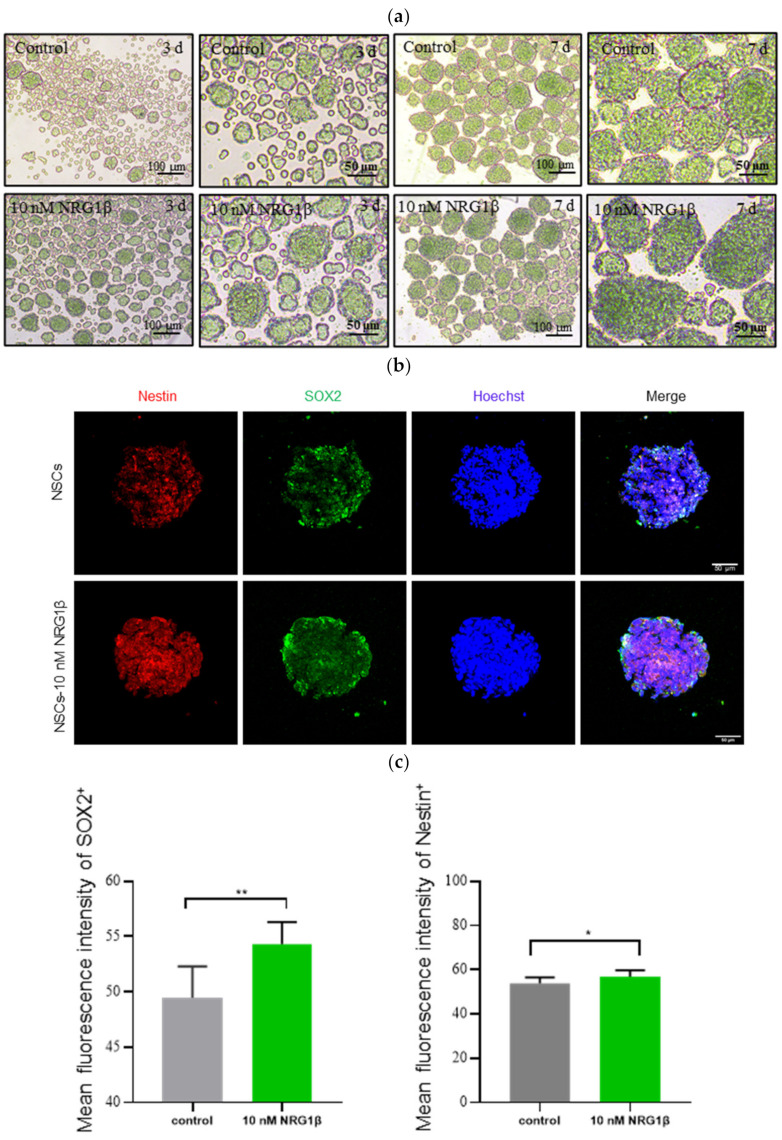

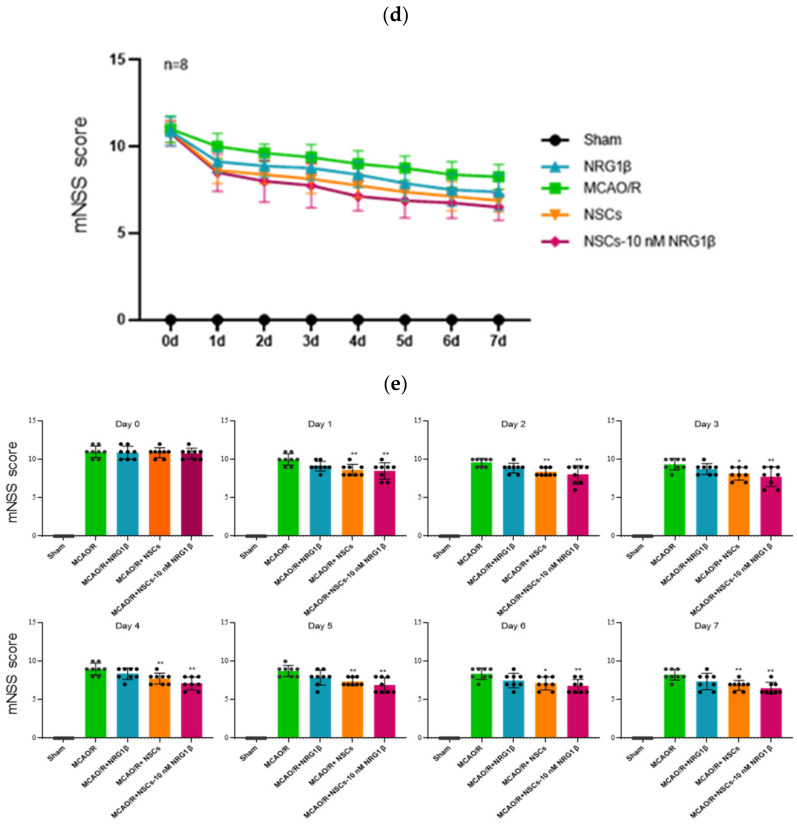
Effects of NSCs transplantation on neurobehavioral score in MCAO/R rats. (**a**) Morphological images of NSCs and NSCs-10 nM NRG1β induced at day 3 and 7 in vitro; (**b**) NSCs is positive for SOX2 (green) and Nestin (red). (**c**) The fluorescence intensity analysis of SOX2 and Nestin in control and 10 nM NRG1β group. (**d**,**e**) Behavioral tests after surgery or transplantation in rats from 0–7 days in MCAO/R, MCAO/R + NRG1β, MCAO/R + NSCs and MCAO/R + NSCs-10 nM NRG1β. Data are expressed as mean ± SD. * *p* < 0.05, ** *p* < 0.01.

In present study, the obtained hUC-MSCs were induced into NSCs with added of growth factors based on the noted method mentioned above (see details in Materials and Methods). As shown in Figure 1a, more and more neurospheres formed when hUC-MSCs were induced into NSCs from the 3rd to 7th day in vitro, and the diameter of the neurospheres gradually increased with the increase of days during induction. Moreover, from the morphological point of view, the efficiency of neurosphere formation in the 10 nM NRG1β group was higher than those in the control group. Importantly, both NSCs and NSCs-10 nM NRG1β is positive for NSCs specific surface antigen markers SOX2 and Nestin (Figure 1b). These data showed that neurospheres have the characteristics of NSCs. Additionally, the fluorescence intensity analysis results showed that the fluorescence intensity of SOX2 and Nestin increased significantly in the 10 nM NRG1β group compared with the control group (Figure 1c, *p* < 0.01, *p* < 0.05). Further, in order to investigate the effects of NSCs pretreated with 10 nM NRG1β transplantation on behavioral dysfunction caused by MCAO/R, the behavioral tests were performed with the mNSS method on day 0 (evaluate the success of the model) and after MCAO/R 1–7 day (Figure 1d,e). Scores in all groups showed a downward trend from day 1 to day 7, but there was no change in the Sham group. Besides, rats with NRG1β, NSCs and NSCs-10 nM NRG1β treatment groups exhibited lower scores than that of MCAO/R group, especially the difference is significant in NSCs and NSCs-10 nM NRG1β transplantation group (*p* < 0.01, Table 3). All these results suggested that transplantation of NSCs and NSCs-10 nM NRG1β showed better effect in enabling neurofunctional recovery in MCAO/R rats.

### 3.2. Transplantation of NSCs Pretreated with NRG1β DECREASE Infarct Volumes and Damage of Cortical Neuron

**Figure 2:**
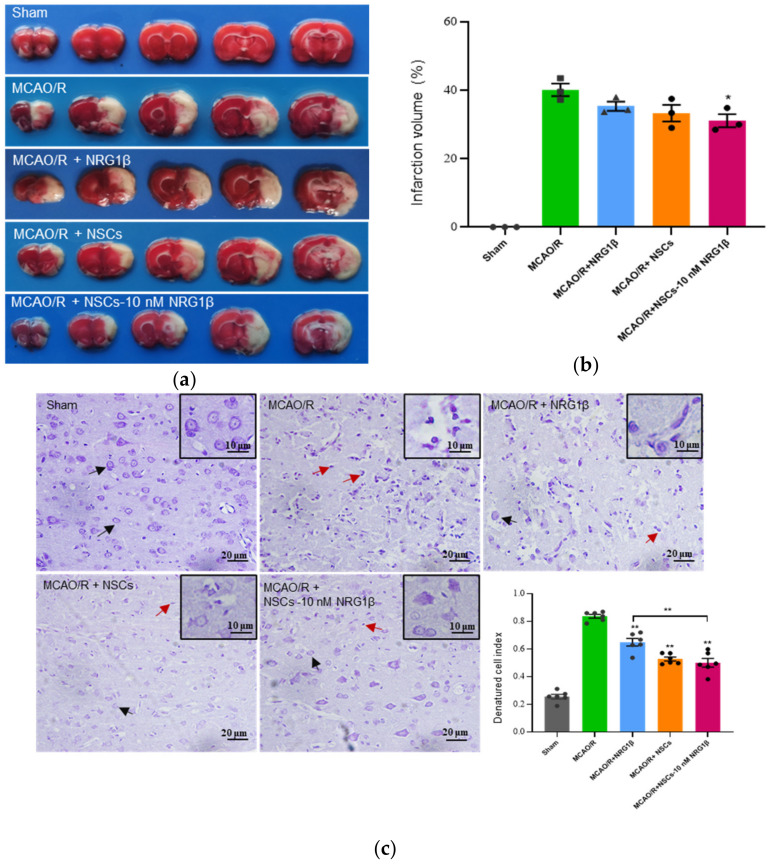
Effect of NSCs-10 nM NRG1β transplantation on infarct volumes and damage of cortical neuron in rats. (**a**) Representative images of infarct volumes in different groups by TTC staining (The white color illustrates the infarct region and red-colored indicate the normal portion). (**b**) The percentage of infarct volume relative to the whole brain of different groups (**c**) Representative morphology of infarct neurons in different groups by Nissl staining. (The black arrow indicates normal cells and the red arrow indicates DCI). DCI in Sham, MCAO/R, MCAO/R + NRG1β, MCAO/R + NSCs and MCAO/R + NSCs-10 nM NRG1β group. Experiments were repeated at least three times, and at least 5 horizons were chosen each time. * *p* < 0.05, ** *p* < 0.01.

In this study, the infarct sizes were assessed by TTC staining on the 7th day after surgery or transplantation (Figure 2a). The infarct volume caused by MCAO/R injury was significantly reduced in the MCAO/R + NSCs-10 nM NRG1β group relative to that of the MCAO/R group (*p* < 0.05), but the reduce was not obvious in NRG1β and NSCs group (Figure 2b). These data showed that NSCs-10 nM NRG1β intervention had a better effect on the improvement of infarct volume than NRG1β and NSCs treatment alone. Nissl staining showed that cortical neurons were orderly and evenly stained, and the structures of the Nissl bodies were complete and the outline was clear on the 7th day in the Sham group. However, the neurons were disordered, the Nissl bodies were shrunken and the staining was deepened in the MCAO/R group. Part of the Nissl bodies were stained normally and had clear contour after NRG1β treatment, and neurons in the NSCs and NSCs-10 nM NRG1β transplantation group had relatively clearer contours, more regular arrangement, and uniform staining compared with the MCAO/R group. Further, the result of DCI analysis showed that the DCI values of NRG1β, NSCs and NSCs-10 nM NRG1β groups were significantly decreased compared with that of the MCAO/R group, an especially more significant effect was observed in the NSCs-10 nM NRG1β group compared with the NRG1β group (Figure 2c, *p* < 0.01).

### 3.3. Transplantation of NSCs Pretreated with NRG1β Change the Expression of Ferroptosis-Related Index in the Cerebral Cortex

**Figure 3:**
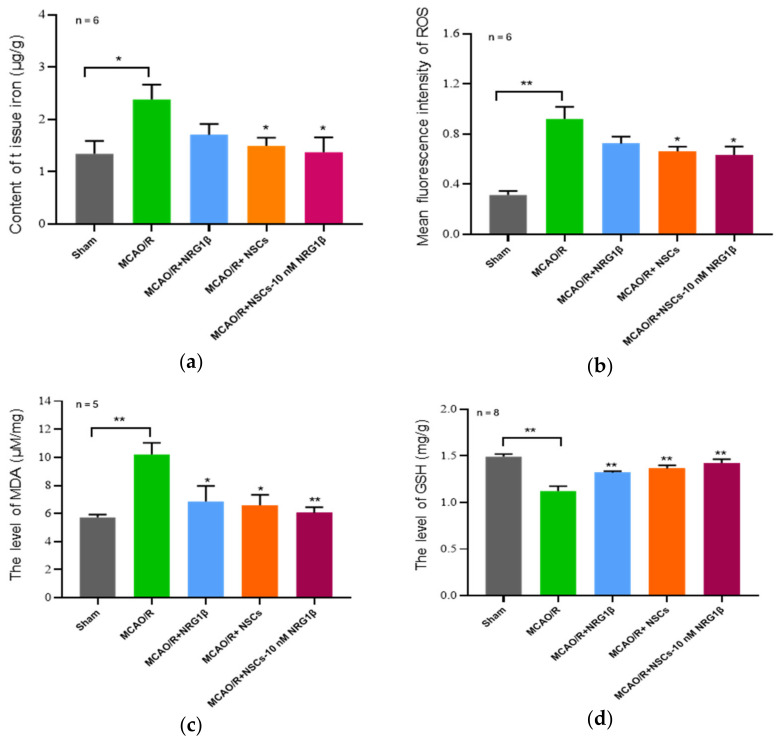
Effect of NSCs transplantation on ferroptosis indicators in ischemic cortex. (**a**) Contents of tissue iron (μg/g) in Sham, MCAO/R, MCAO/R+ NRG1β, MCAO/R+NSCs, MCAO/R+ NSCs-10 nM NRG1β groups. (**b**) The level of ROS in different groups. (**c**,**d**) The expression of MDA and GSH in different groups. * *p* < 0.05, ** *p* < 0.01.

The levels of Fe^2+^, ROS, MDA and GSH in the cerebral cortex were measured in the Sham, MCAO/R, NRG1β, NSCs and NSCs-10 nM NRG1β groups after intervention for 7 days. As shown in Figure 3a–c, the levels of Fe^2+^, ROS and MDA were significantly increased in the MCAO/R group compared with the Sham group (*p* < 0.05 or *p* < 0.01), but decreased after intervention by NRG1β, NSCs and NSCs-10 nM NRG1β; and the difference is more obvious in NSCs and NSCs-10 nM NRG1β than in the NRG1β group (*p* < 0.05 or *p* < 0.01). Particularly, NSCs-10 nM NRG1β had the most significant improvement effect compared with other groups in terms of the level of MDA (*p* < 0.01). Besides, the content of GSH in MCAO/R group was significantly lower than that of Sham group, while the treatment with NRG1β, NSCs and NSCs-10 nM NRG1β was significantly higher than that of the MCAO/R group respectively (*p* < 0.01).

### 3.4. Transplantation of NSCs Pretreated with NRG1β Alleviates Mitochondrial Damage in Cerebral Cortex Neurons

**Figure 4:**
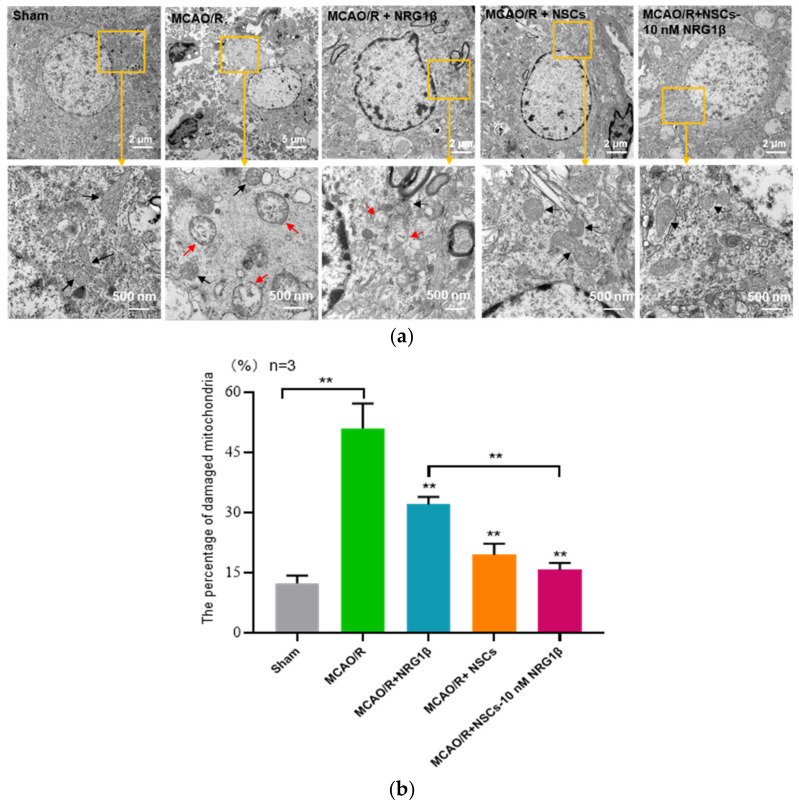
Effect of NSCs transplantation on mitochondrial injury in ischemic cortical region. (**a**) Representative pictures of neuron mitochondrial morphological structure in each group; red arrows indicate damaged mitochondria, and black arrows indicate normal mitochondria. (**b**) Percentage of damaged mitochondria. ** *p* < 0.01.

The change of mitochondrial morphology is another significant feature of ferroptosis. Therefore, we analyzed the ultrastructure mitochondrial of neurons in the ischemic cortex. In the Sham group, the morphological structure and numbers of mitochondria were normal. In the MCAO/R group, many mitochondrial intimae ruptured, and mitochondrial cristae fractured or disappeared. Specially, after NRG1β, NSCs and NSCs-10 nM NRG1β intervention, the degree of neuron injury was alleviated to a certain extent, and the mitochondrial intima integrity was improved, the mitochondrial crest structure was clearer, and the overall mitochondrial injury was rescued (Figure 4a). Furthermore, statistical results showed that the percentage of damaged mitochondria was obviously increased compared with the Sham group (*p* < 0.01), and decreased after NRG1β, NSCs and NSCs-10 nM NRG1β intervention (*p* < 0.01); Importantly, the difference was significant between NRG1β and NSCs-10 nM NRG1β group (Figure 4b, *p* < 0.01).

### 3.5. Transplantation of NSCs Pretreated with NRG1β Affects Ferroptosis in Cerebral Cortex of Rats

**Figure 5:**
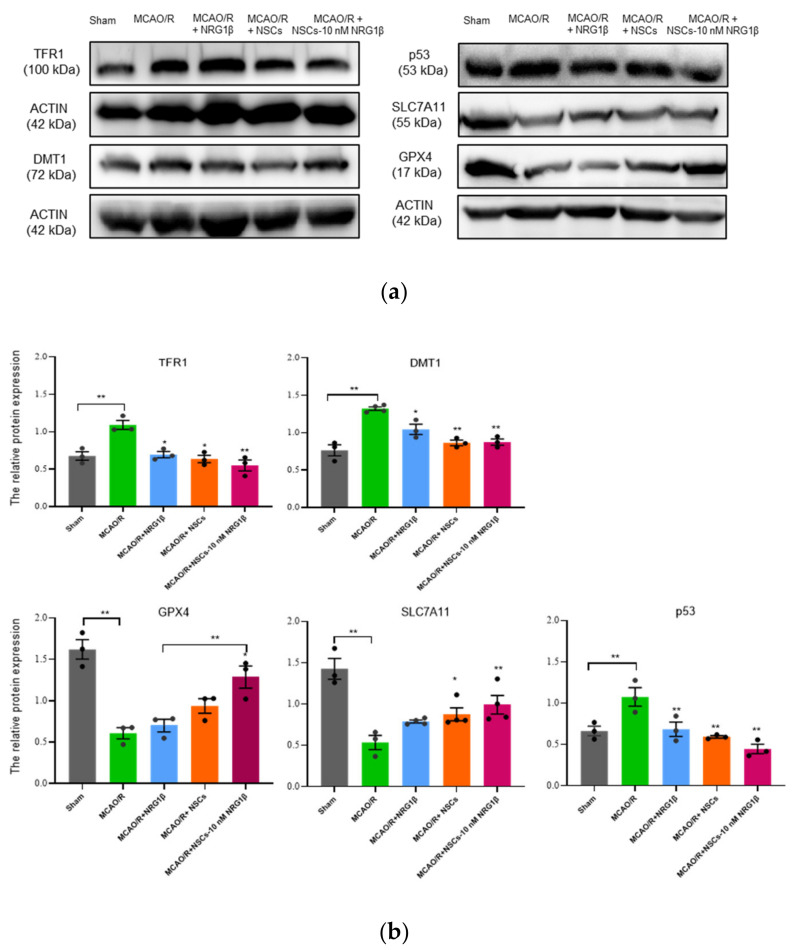
Effect of NSCs transplantation on expression of ferroptosis related proteins in ischemic cortex. (**a**) The expression levels of DMT1, TFR1, p53, SLC7A11 and GPX4 protein detected by Western blot. (**b**) Gray value analysis of DMT1, TFR1, p53, SLC7A11 and GPX4, and the protein expression levels in different groups. * *p* < 0.05, ** *p* < 0.01.

Further, based on the results above, the key proteins related to ferroptosis in the ischemic cortex were detected in each group. Western blot showed that the TFR1 and DMT1 proteins in the MCAO/R group were significantly higher than that of the Sham group (*p* < 0.01), while it was decreased after NRG1β, NSCs and NSCs-10 nM NRG1β intervention. At the same time, the most significant effect was observed in the NSCs and NSCs-10 nM NRG1β group in terms of the expression of DMT1, but the NSCs-10 nM NRG1β group is most significant in the expression of TFR1 (Figure 5a,b, *p* < 0.01). Additionally, the expression levels of GPX4 and SLC7A11 in the Sham group were significantly higher than that of the MCAO/R group (*p* < 0.01), and after NRG1β, NSCs and NSCs-10 nM NRG1β intervention, the expressions of GPX4 and SLC7A11 were increased compared with the MCAO/R group. However, the difference in NSCs-10 nM NRG1β group was the most significant compared with the MCAO/R group. (*p* < 0.05 or *p* < 0.01); In addition, the expression of p53 in the MCAO/R group was significantly higher than that of the Sham group (*p* < 0.01), which was decreased in all groups after treatment with NRG1β, NSCs and NSCs-10 nM NRG1β (*p* < 0.01).

## 4. Discussion

Research indicates that stem cells transplantation is gradually becoming a new therapy for cerebral ischemia reperfusion injury [37]. Among them, NSCs have become an ideal candidate cell type for the treatment of cerebral ischemia reperfusion injury due to their excellent self-renewal ability and multi-directional differentiation potential. Particularly, they can differentiate into multiple brain cells types [38,39]. However, the technical difficulties associated with the isolation and proliferation of NSCs, low survival rate and differentiation into neuronal after transplantation limited their potential for clinical applications. At the same time, their efficacy is also limited in the ischemic microenvironment rich in ROS, inflammatory factors, and excitatory amino acids [10]. Therefore, it is of great importance to find an alternative source of NSCs to promote their adaptability in the brain histopathological environment and effectively improve their functional recovery in damaged brain tissues.

According to reports, MSCs can differentiate into NSCs or neural progenitor cells (NPCs) under certain conditions [40,41]. Based on this, hUC-MSCs were isolated and obtained in this study, and then NSCs were formed through the growth factors induction method. Moreover, studies have confirmed that bFGF and EGF are key inducers of differentiation from MSCs to NSCs, and the survival time of NSCs induced by growth factors is significantly longer than that of those induced by chemical induction [42]. Therefore, the medium supplemented with 20 ng/mL EGF and 20 ng/mL bFGF was used as the differentiation medium for hUC-MSCs to NSCs in this study. 

Currently, much of the research about NSCs therapy is focused on exploring new methods to improve the therapeutic efficiency. It has been reported that viral vectors and gene editing NSCs may trigger potential safety problems, while the cytokines are innocuous, which can regulate the self-renewal, proliferation, and differentiation of stem cells, as well as the survival microenvironment of stem cells, and maximize their therapeutic potential [11]. Studies have shown that the combined administration of vascular endothelial growth factor (VEGF) and human NSCs has a certain synergistic effect on the treatment of stroke, and the combined treatment group showed better behavioral recovery and significantly reduced cerebral atrophy in the cerebral cortex and striatum than the single treatment group [43]. In addition, co-delivery of IFN-γ (50 ng) enhanced the role of transplanted NSCs in ischemic rats. The combined treatment group was found to have significantly increased neurogenesis compared to other groups in vivo [44]. It is also reported that co-transplantation of vascular progenitor cells (VPC) and NSCs improved the differentiation rate of neurons and the formation of microvessels in vivo, significantly improved motor function and reduced infarct volume in cerebral ischemia rats [45]. Importantly, previous studies in our laboratory have demonstrated that NRG1β can rescue cerebral ischemia/reperfusion injury [26]. Based on the above studies, NRG1β was added during the induction of hUC-MSCs to NSCs, and MCAO/R model was used to further investigate the neuroprotective effect and the possible molecular mechanism of NRG1β pretreated NSCs on ischemia reperfusion injury in vivo.

Our research finds that NRG1β, NSCs and NSCs-10 nM NRG1β intervention improved behavioral scores to a certain extent compared with MCAO/R group on the 7th day, and NSCs and NSCs-10 nM NRG1β group had the most significant effect among them according to difference analysis compared with MCAO/R group, but the difference is not significant between NSCs and the NSCs-10 nM NRG1β group. Besides, results showed that NSCs-10 nM NRG1β had significant difference in improving infarct volume, while the other two groups had no significant difference. Likewise, the degree of damaged neurons in the ischemic cortex also improved in certain degree in all three intervention groups compared with MCAO/R group but there was no significant difference among the three intervention groups. Meanwhile, in terms of Fe^2+^ and ROS levels, the improvement effect of NSCs and NSCs-10 nM NRG1β on MCAO/R rats are significantly better than those of NRG1β, but this improvement effect is not significant between NSCs and NSCs-10 nM NRG1β group. However, the improvement effect on MDA was the most significant in the NSCs-10 nM NRG1β group among the three intervention groups. Further, detection of mitochondrial injury showed that NRG1β, NSCs and NSCs-10 nM NRG1β intervention could alleviate mitochondrial injury caused by MCAO/R, and the improvement effect was significant between NSCs-10 nM NRG1β and NRG1β group but not obviously between NSCs and NSCs-10 nM NRG1β group. Based on the results above, we can find that although NRG1β, NSCs and NSCs-10 nM NRG1β intervention played a certain protective role in the damage caused by MCAO/R and there do not seem to be any significant differences between the NSCs and NSCs-10 nM NRG1β group in terms of some indexes, the intervention effect of NSCs-10 nM NRG1β group still is better than that of the NRG1β and NSCs group overall in terms of infarct volume and the level of MDA, meanwhile, intervention group could influence ferroptosis related index after ischemia reperfusion injury. Based on this, we speculated that the pretreatment of NSCs with 10 nM NRG1β may enhance the adaptability of NSCs to the ischemic microenvironment, so that the pretreatment group has a potential marginal improvement than the NSCs alone. 

After ischemic stroke, the blood–brain barrier is destroyed and excessive Fe^3+^ in blood is released into the brain parenchyma via transferrin (TF) and TFR1. In the presence of six-transmembrane epithelial antigen of prostate 3 (STEAP3), DMT1 transport Fe^3+^ from endosomes as Fe^2+^ to cytoplasm [46]. Due to the change of Fe^2^^+^ content in injury tissue, our research further confirmed that there was also a difference in the expression of TFR1 and DMTI in intervention groups. This data suggested that the intervention group may change iron metabolism by influencing the expression of TFR1 and DMTI, thus leading to the occurrence of ferroptosis. Additionally, studies have reported that SLC7A11 mainly plays a role in the transformation of extracellular cysteine and intracellular glutamate. The down-regulation of this protein leads to the imbalance of intracellular glutamate and further induces the inactivation of GPX4, which is involved in inhibiting lipid peroxidation and ROS recruitment, ultimately resulting in ferroptosis [47,48,49]. Also, SLC7A11, as a direct target of p53, can negatively regulate the expression of p53, thereby reducing extracellular cysteine uptake and intracellular GSH synthesis and activating cell ferroptosis [50,51]. Based on this, we detected the expressions of p53, GPX4 and SLC7A11 in Sham, MCAO/R and NRG1β, NSCs and NSCs-10 nM NRG1β intervention groups. Our studies showed that the levels of GPX4 and SLC7A11 increased after NRG1β, NSCs and NSCs-10 nM NRG1β intervention compared with MCAO/R, and the NSCs-10 nM NRG1β showed the most significant difference among the three groups. On the contrary, the level of p53 was decreased. These data are suggestive to some extent that ferroptosis maybe a key pathway involved in the improvement of NSCs pretreated with NRG1β in ischemic reperfusion injury.

## 5. Conclusions

This study suggested that NSCs pretreated with NRG1β and NSCs had a better effect than NRG1β treated alone on the improvement on damage caused by MCAO/R in a certain extent, especially there was a potential marginal improvement of NSCs pretreated with NRG1βon the damage caused by MCAO/R compared with NSCs and NRG1β treated alone, which provides a new idea for improving the effect of NSCs transplantation on cerebral ischemia reperfusion injury. In addition, studies also suggested that the intervention may play a neuroprotective role by influencing iron metabolism and activating p53/SLC7A11/GPX4 to regulate ferroptosis, which also provides a new direction for improving the therapeutic effect of stem cells transplantation in cerebral ischemia reperfusion injury.

## Figures and Tables

**Table 1:** Modified Neurological Severity Score (mNSS).

Items		Scores
Raising rat by tail	Flexion of forelimb	1
	Flexion of hindlimb	1
	Head moved >10° to vertical axis within 30 s	1
Placing rat on floor (normal = 0, maximum = 3)	Normal walk	0
	Inability to walk straight	1
	Circling toward paretic side	2
	Falls to paretic side	3
Sensory tests	Placing test (visual and tactile test)	1
	Proprioceptive test (deep sensation, pushing paw against table edge to stimulate limb muscles)	1
Beam balance tests (normal = 0; maximum = 6)	Balances with steady posture	0
	Grasps side of beam	1
	Hugs beam and one limb fall from beam	2
	Hugs beam and two limbs fall from beam, or spins on beam (>60 s)	3
	Attempts to balance on beam but falls off (>40 s)	4
	Attempts to balance on beam but falls off (>20 s)	5
	Falls off; no attempt to balance or hang on to beam (<20 s)	6
Reflex absence and abnormal movements	Pinna reflex (head shake when auditory meatus is touched)	1
	Corneal reflex (eye blink when cornea is lightly touched with cotton)	1
	Startle reflex (motor response to a brief noise from snapping a clipboard paper)	1
	Seizures, myoclonus, myodystonia	1

**Table 2:** Primary antibodies.

Antibody	Vendor	Cat. No	Dilution	Applications
ACTIN	Proteintech	20536-1-AP	1:1000	Western blot
GPX4	ZENBIO	381958	1:1000	Western blot
SLC7A11	ABclonal	A15604	1:1000	Western blot
p53	ABclonal	A3185	1:1000	Western blot
TFR1	ABclonal	A10231	1:1000	Western blot
DMT1	ABclonal	A5865	1:1000	Western blot

**Table 3:** mNSS score (Mean ± SD).

	Sham	MCAO/R	MCAO/R + NRG1β	MCAO/R + NSCs	MCAO/R + NSCs- 10 nM NRG1β
Day 0	0.00 ± 0.00	11.00 ± 0.76	10.88 ± 0.83	10.88 ± 0.64	10.75 ± 0.71
Day 1	0.00 ± 0.00	10.00 ± 0.76	9.13 ± 0.64	8.63 ± 0.74	8.50 ± 1.07
Day 2	0.00 ± 0.00	9.63 ± 0.52	8.88 ± 0.64	8.37 ± 0.52	8.00 ± 1.20
Day 3	0.00 ± 0.00	9.38 ± 0.74	8.75 ± 0.71	8.13 ± 0.83	7.75 ± 1.28
Day 4	0.00 ± 0.00	9.00 ± 0.75	8.38 ± 0.74	7.75 ± 0.71	7.13 ± 0.83
Day 5	0.00 ± 0.00	8.75 ± 0.71	7.88 ± 0.99	7.38 ± 0.52	6.88 ± 0.99
Day 6	0.00 ± 0.00	8.38 ± 0.74	7.50 ± 0.93	7.13 ± 0.83	6.75 ± 0.89
Day 7	0.00 ± 0.00	8.25 ±0.71	7.38 ± 1.06	6.88 ± 0.64	6.50 ± 0.76

## Data Availability

The data in this study could be available from the corresponding author.
